# Strand Displacement Activity of PrimPol

**DOI:** 10.3390/ijms21239027

**Published:** 2020-11-27

**Authors:** Elizaveta O. Boldinova, Ekaterina A. Belousova, Diana I. Gagarinskaya, Ekaterina A. Maltseva, Svetlana N. Khodyreva, Olga I. Lavrik, Alena V. Makarova

**Affiliations:** 1 Institute of Molecular Genetics, National Research Center “Kurchatov Institute”, Kurchatov sq. 2, 123182 Moscow, Russia; lizaboldinova@yandex.ru (E.O.B.); deanaw@yandex.ru (D.I.G.); 2 Institute of Chemical Biology and Fundamental Medicine, Siberian Branch of the Russian Academy of Sciences, 8 Lavrentiev Avenue, 630090 Novosibirsk, Russia; sheffield@ngs.ru (E.A.B.); 060179@mail.ru (E.A.M.); svetakh@niboch.nsc.ru (S.N.K.); lavrik@niboch.nsc.ru (O.I.L.)

**Keywords:** PrimPol, strand displacement, RPA, PolDIP2, FEN1

## Abstract

Human PrimPol is a unique enzyme possessing DNA/RNA primase and DNA polymerase activities. In this work, we demonstrated that PrimPol efficiently fills a 5-nt gap and possesses the conditional strand displacement activity stimulated by Mn^2+^ ions and accessory replicative proteins RPA and PolDIP2. The DNA displacement activity of PrimPol was found to be more efficient than the RNA displacement activity and FEN1 processed the 5′-DNA flaps generated by PrimPol in vitro.

## 1. Introduction

Human PrimPol is an archaeo-eukaryotic primase with DNA polymerase activity found in nuclei and mitochondria [1,2]. Unlike the complex of Pol α-primase possessing RNA primase and DNA polymerase activities, PrimPol demonstrates DNA primase activity [1,2]. PrimPol also possesses an ability to anneal single stranded DNA (ssDNA) molecules based on microhomology and connect separate strands [3]. It has been suggested that the main function of PrimPol is re-initiation of stalled replication forks using de novo DNA synthesis and re-priming downstream DNA lesions and non-B DNA structures such as G-quadruplexes [4,5,6,7]. PrimPol also possesses DNA translesion activity. PrimPol efficiently and relatively accurately bypasses 8-oxoguanine and other non-bulky DNA lesions [2,8].

During DNA repair and replication, DNA polymerases often carry out strand displacement DNA synthesis. Several human DNA polymerases possess the strand displacement activity: Pol δ [9,10], Pol β [11,12,13], Pol η [10,14,15,16,17], Pol κ [15], as well as yeast Pol ζ [10,15,16], whereas Pol ε, Pol α, and Pol γ have limited ability for strand displacement and displace only 1–2 nucleotides [14,18,19]. Many polymerases require accessory proteins, such as DNA helicases, to carry out strand displacement synthesis [20]. In cells, DNA polymerases might polymerize across a gap. Moreover, the presence of a 5′-phosphorylated downstream strand is critical for the highest catalytic efficiency of some DNA polymerases, such as Pol β and Pol λ [21].

The activity of PrimPol on DNA substrates with a gap and the ability of PrimPol to displace DNA strands has not been studied yet. In this article, we investigated how PrimPol responds to a gapped DNA structure and analyzed its ability to displace downstream DNA or RNA strands. We demonstrated that PrimPol possesses the DNA strand displacement activity, which is stimulated by Mn^2+^ ions, replication protein A (RPA) and polymerase delta-interacting protein 2 (PolDIP2), and that the resulting flap can be subsequently removed by FEN1.

## 2. Results

### 2.1. Mn^2+^ Ions Stimulate the DNA Strand Displacement Activity of PrimPol

**Figure 1:**
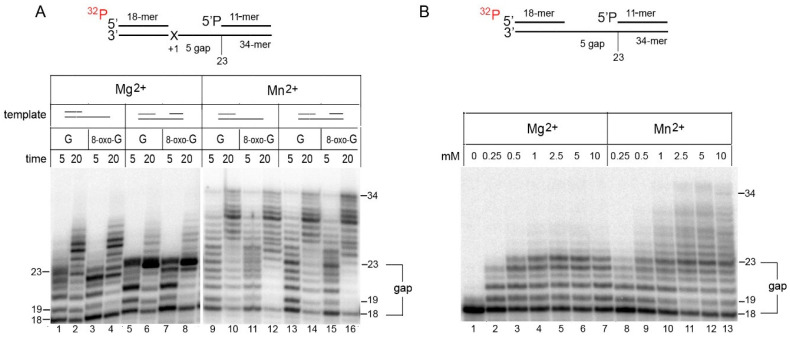
The strand displacement activity of PrimPol on DNA substrates with the 5-nt gap. (**A**) Strand displacement synthesis of PrimPol on a 34-mer DNA substrate. Primer extension reactions were carried out on DNA substrates with the 5-nt gap or with the 3′-recessed end in the presence of 1 mM Mg^2+^ or 3 mM Mn^2+^ and 400 nM PrimPol for 10 min. The DNA template contains undamaged G or 8-oxoguanine (8-oxo-G) in the +1 position downstream of the primer. Denaturing urea-PAGE of gap-filling products is shown. (**B**) The effect of different Mg^2+^ and Mn^2+^ ion concentrations on the strand displacement synthesis by PrimPol. Primer extension reactions were carried out on a 34-mer undamaged DNA substrate with the 5-nt gap in the presence of 0.25–10 mM Mg^2+^ or Mn^2+^ and 100 nM PrimPol for 10 min. Denaturing urea-PAGE of gap-filling products is shown.

PrimPol utilizes both Mg^2+^ and Mn^2+^ ions. Mn^2+^ ions significantly stimulate the DNA polymerase and DNA primase activities of PrimPol but decrease the accuracy of dNMP incorporation [2,22,23]. We probed the DNA polymerase activity of PrimPol on a gapped DNA substrate in Mg^2+^ and Mn^2+^ reactions (Figure 1). The 34-mer DNA substrate contained the 18-mer primer, the 5-nt gap with G or 8-oxoguanine (8-oxo-G) lesion in the +1 position, and the 11-mer locking DNA strand with the 5′-phosphate. Simple primer extension reactions on DNA substrates without locking DNA strands were used as controls in parallel, to monitor the “pausing” and efficiency of the strand displacement activities analyzed (Figure 1A, lanes 1–4 and 9–12). PrimPol efficiently filled the gap containing undamaged G or 8-oxo-G (Figure 1A). Only trace DNA strand displacement activity of PrimPol was observed in the presence of Mg^2+^ ions (Figure 1A, lanes 5–8). In contrast, PrimPol was capable of displacing the 11-mer DNA oligonucleotide to make a 34 bp duplex DNA in the presence of Mn^2+^ ions (Figure 1B, lanes 13–16).

We also analyzed the ability of PrimPol to displace DNA in the presence of different Mg^2+^ and Mn^2+^. Processive DNA synthesis and the robust displacement of the 11-mer DNA strand by PrimPol were supported by 2.5–10 mM MnCl_2_ (Figure 1B, lanes 11–13). A small amount of the 23-nt gap-filled product was accumulated, indicating a pause site and/or suggesting that the efficiency of DNA strand displacement synthesis is lower than gap-filling.

**Figure 2:**
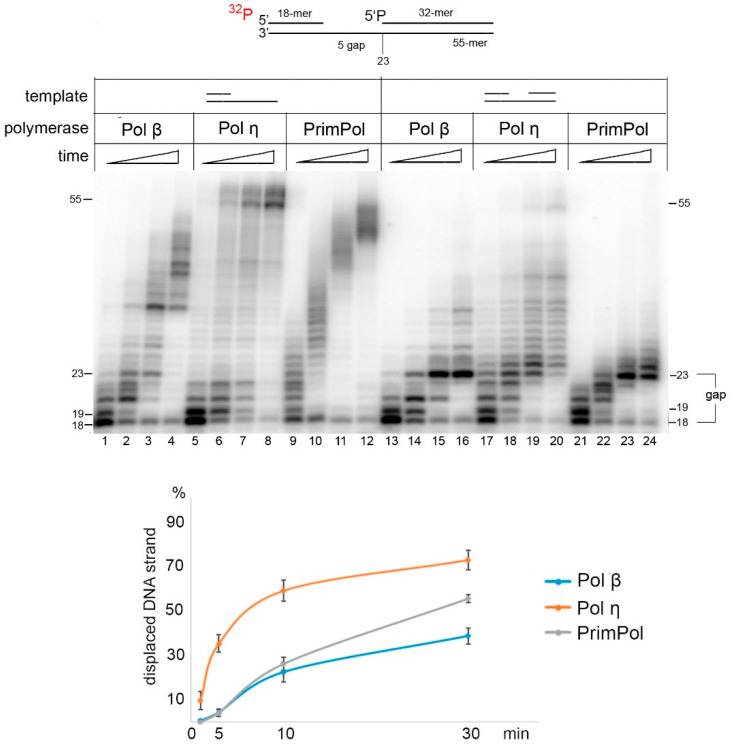
Comparison of DNA strand displacement synthesis between human Pol β, Pol η, and PrimPol. Primer extension reactions were carried out on a 55-mer DNA substrate with the 5-nt gap in the presence of 10 mM Mg^2+^ and 10 nM Pol β or Pol η, or in the presence of 1 mM Mn^2+^ and 100 nM PrimPol for 1–30 min.

Next, we analyzed the DNA strand displacement activity of PrimPol in reactions with low Mn^2+^ concentrations and long locking DNA/RNA strands. We tested the DNA strand displacement activity of PrimPol on the 55-mer DNA substrate with the 32-mer locking DNA in the presence of 1 mM Mn^2+^ (Figure 2). In these conditions, the DNA strand displacement activity was relatively weak and only displacement of a few nucleotides was observed. The efficiency of DNA strand displacement by PrimPol was inferior to that of Pol η (Figure 2, lanes 17–20) and comparable to Pol β (Figure 2, lanes 13–16).

### 2.2. RNA Strand Displacement Activity of PrimPol Is Not Efficient

**Figure 3:**
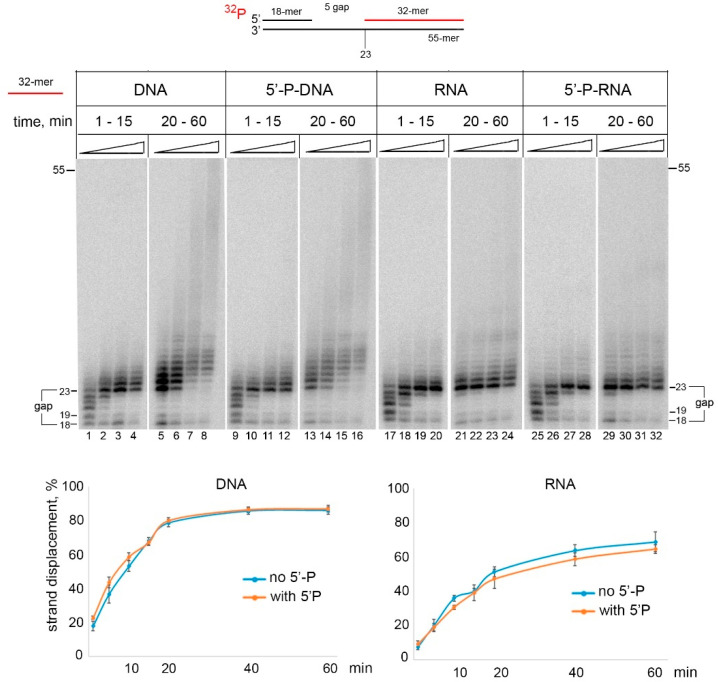
Characterization of PrimPol strand displacement activity. Primer extension reactions were carried out on DNA substrates with the gap containing 5′-phosphorylated or non-phosphorylated downstream DNA or RNA in the presence of 1 mM Mn^2+^ and 100 nM PrimPol for 1–60 min. The percent of displaced primer (from +5 pause) was calculated for each reaction, and the mean values of displaced primers with the standard errors are shown on the diagrams.

We also tested whether PrimPol is able to displace downstream RNA and analyzed the effect of the 5**′**-phosphate of the locking DNA/RNA strand (Figure 3). The RNA displacement activity of PrimPol was significantly less efficient than the DNA displacement activity (Figure 3, lanes 1–8 and 17–24). The 5**′**-phosphate had no effect on the DNA and RNA displacement synthesis by PrimPol (Figure 3, lanes 9–16 and lanes 25–32).

### 2.3. FEN1 Processes the 5′-Flaps Generated by PrimPol In Vitro

**Figure 4:**
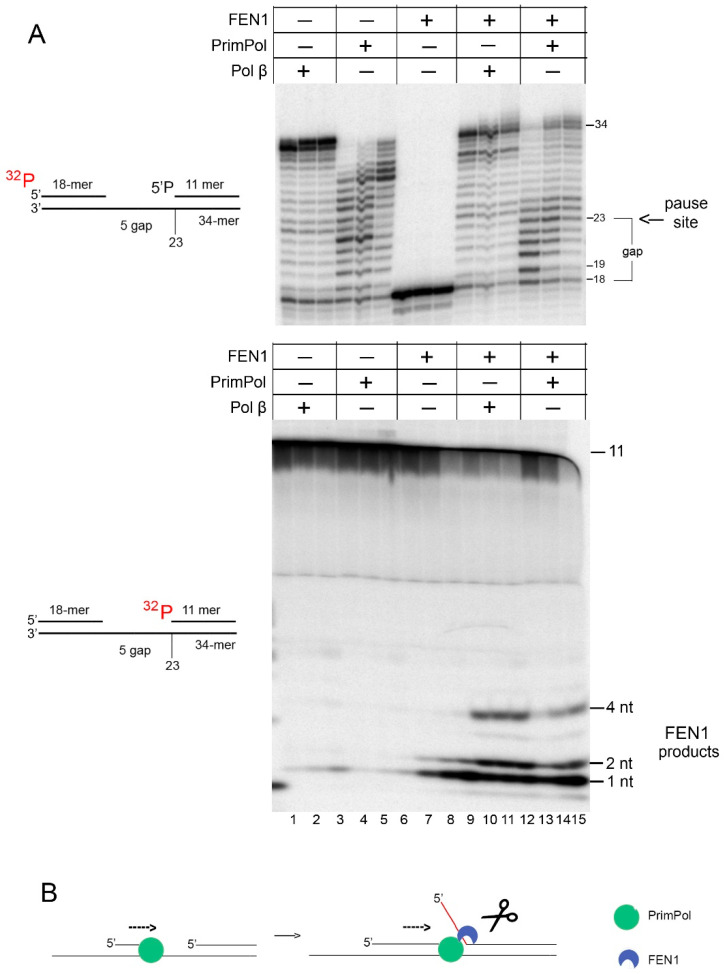
(**A**) Processing of PrimPol-generated 5′-flaps by FEN1. Primer extension reactions were carried out on the 32-mer DNA substrate with the gap containing 5′-^32^P-labeled 18-mer DNA primer or 5′-^32^P-labeled 11-mer locking DNA in the presence of 400 nM FEN1, 3 mM Mn^2+^, and 100 nM PrimPol for 5–30 min. Probable lengths of FEN1 products are shown on the gel. (**B**) The scheme of the 5′-flap cleavage by FEN1.

Endonuclease FEN1 excises 5**′**-flap structures in DNA during the long-patch BER [13,24,25] and Okazaki fragment maturation [26]. We labeled the locking DNA strand at the 5**′**-end with 32P and monitored its electrophoretic mobility (Figure 4, bottom panel). FEN1 cleaved the 2–4 nt 5**′**-flaps created by Pol β (Figure 4, lanes 10–12) and PrimPol (Figure 4, lanes 13–15), and the 5**′**-3**′**-exonuclease products of FEN1 (1 nt and 2 nt) were also observed in all reactions with FEN1 (Figure 4, lanes 7–15) [27]. In the presence of Mn^2+^ ions, PrimPol carried out more efficient DNA synthesis and/or strand displacement activity when FEN1 was added to the reaction. FEN1 eliminated the pausing observed during displacement of the locking DNA strand at the 23 nt position of the DNA substrate.

### 2.4. The Strand Displacement Activity of PrimPol Is Stimulated by PolDIP2 and RPA

**Figure 5:**
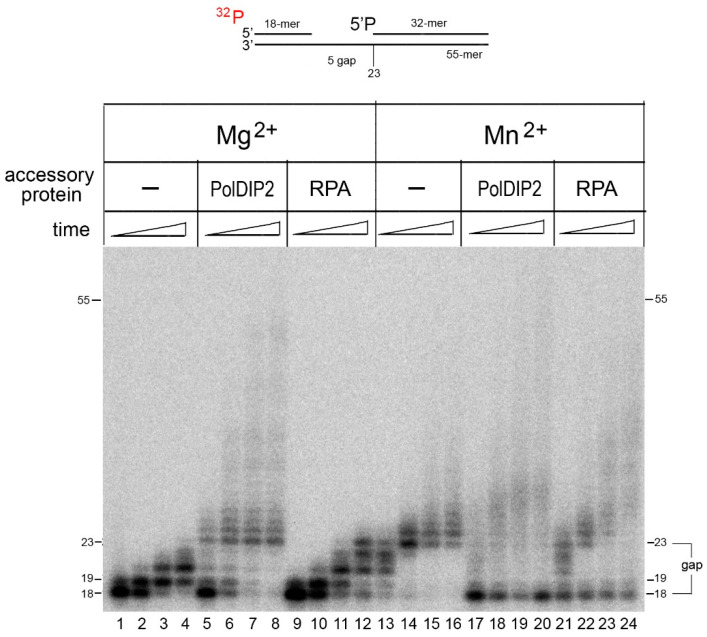
The effect of PolDIP2 and RPA on the DNA strand displacement activity of PrimPol. Primer extension reactions were carried out on DNA substrates with the gap containing 5′-phosphorylated downstream DNA in the presence of 10 mM Mg^2+^ or 1 mM Mn^2+^ and 100 nM PrimPol for 1–20 min.

We also analyzed the DNA strand displacement activity of PrimPol in the presence of PolDIP2 and RPA accessory proteins. Previously, it was shown that PolDIP2 and RPA functionally interact with PrimPol and stimulate its DNA polymerase activity [28,29,30]. In agreement with these studies, PolDIP2 significantly stimulated the DNA strand displacement activity of PrimPol on the DNA substrate with a gap in the presence of Mn^2+^ ions (Figure 5, lanes 17–20). Importantly, PrimPol demonstrated the DNA strand displacement activity in the presence of PolDIP2 (but not RPA), even in Mg^2+^-reaction conditions (Figure 5, lanes 5–8). The stimulation of strand displacement synthesis by RPA (under equimolar RPA to DNA ratios) was observed only in the presence of Mn^2+^ ions (Figure 5, lanes 21–24).

## 3. Discussion

In this work, we demonstrated that human PrimPol efficiently fills the 5-nt gap and possesses the conventional DNA strand displacement activity in the presence of Mn^2+^ but not Mg^2+^ ions. The presence of the 5′-phosphate on the downstream DNA strand did not increase the activity of PrimPol on gapped DNA. These data are consistent with the role of Mn^2+^ ions in the stimulation of the DNA polymerase and primase activities of PrimPol, as well as the stimulation of DNA damage bypass [2,8,22,23]. Efficient gap-filling synthesis was also observed on gapped DNA with 8-oxo-G lesion. Efficient 8-oxo-G bypass on DNA substrates with 5′-recessed ends was demonstrated previously [2,8,23].

In cells, strand displacement activity of PrimPol can be stimulated by replication accessory factors, such as proteins recruiting PrimPol to DNA and enzymes involved in a 5′-flap cleavage. PrimPol does not interact with proliferating cell nuclear antigen (PCNA), but another protein functionally interacting with several DNA polymerases [31]—PolDIP2—stimulates the DNA polymerase activity of PrimPol [28]. The interaction between PrimPol and PolDIP2 was demonstrated by cross-linking and mass spectrometry analysis [28]. RPA recruits PrimPol to the sites of DNA damage and stimulates its DNA polymerase and DNA primase activities [29,30,32]. RPA takes part in the assembly and coordination of the replication fork due to its protein–protein interactions [33]. In this work, we demonstrated that the DNA strand displacement activity of PrimPol can be stimulated by both replicative accessory proteins on a DNA substrate with the 5-nt gap. RPA efficiently binds long ssDNA molecules and shows a stable 30 nt binding mode [34]. However, RPA also binds 4–6 nucleotides [35] and utilizes a ssDNA binding site with the size of 8–10 nucleotides [35,36]. The mode of RPA binding is dependent on the length of the ssDNA platform, as well as on the ratio of RPA:DNA concentration [37]. RPA also contacts flaps in DNA with a short gap [36]. Possibly, the stimulation of the strand displacement activity of PrimPol by RPA can be explained by the physical interaction of polymerase with RPA and its additional attraction to the ssDNA part of the gap from the 5′-side by RPA associated with the flap.

Endonuclease FEN1 excises 5′-flap structures in DNA during the long-patch BER [13,24,25] and stimulates the DNA strand displacement activity of Pol β and Pol λ [11,12,13]. FEN1 also physically interacts with Pol β [12] and plays a role in the long-patch BER in nuclei [13,25] and mitochondria [38]. The complex of Pol δ–PCNA–FEN1 removes RNA primers during Okazaki fragment maturation [26]. Importantly, FEN1 was found among the proteins interacting with PrimPol using mass spectrometry [39]. We showed that PrimPol cooperates with FEN1 to displace and remove downstream DNA strand in vivo (Figure 4B). Possibly, FEN1 can cooperate with PrimPol to perform stepwise strand displacement synthesis in the nucleus and/or mitochondria.

Altogether, our data suggest that PrimPol can realize the DNA strand displacement activity in vivo. However, the functional importance of the strand displacement activity of PrimPol is not clear. The process of strand displacement synthesis is involved in many DNA transactions in vivo, such as d-loop invasion in homologous recombination and break-induced replication, non-homologous end-joining and microhomology-mediated end-joining, long-patch base excision repair, and Okazaki fragment maturation. However, human PrimPol has not been reported to be involved in these processes. The unusual ability of PrimPol to connect separate DNA strands [3], together with the DNA strand displacement activity, opens up the possibility of a role of PrimPol in microhomology-mediated end-joining. Additionally, it is possible that PrimPol in cooperation with nucleases and/or helicases (such as FEN1, Twinkle, or DNA2) might realize the strand displacement activity during re-priming events of mitochondrial DNA replication. Interestingly, the RNA displacement activity of PrimPol was not efficient, suggesting that PrimPol is blocked after encountering an Okazaki fragment.

## 4. Materials and Methods

### 4.1. Proteins

PrimPol was purified as described [40]. The full-length PolDIP2 coding gene was cloned into the pGEX6P1 plasmid in frame with an N-terminal GST-tag. PolDIP2 was expressed at 22 °C after induction with 0.5 mM isopropyl β- d-1-thiogalactopyranoside in 6 L of Rosetta 2 *Escherichia coli* cells and purified as described for PrimPol in three steps: (1) batch binding to 1 mL of glutathione-sepharose resin (GE Healthcare, Chicago, IL, USA), (2) the GST-tag cleavage with 3C protease, and (3) 1 mL HiTrap Heparin HP column (GE Healthcare, Chicago, IL, USA) chromatography. PolDIP2 was eluted at 250 mM KCl, aliquoted and stored at −80 °C. PolDIP2 aliquots were discarded after each experiment to avoid freeze–thaw–refreeze cycles. FEN1 and RPA were purified as described [41,42]. Fractions containing substoichiometric RPA were discarded, and RPA with ~1:1:1 stoichiometry of RPA1:RPA2:RPA3 subunits was used in experiments. Preparations of PolDIP2, FEN1, and RPA (Supplementary Materials Figure S1) were free of contaminating DNA polymerase activity.

### 4.2. DNA Substrates

Oligonucleotide substrates and primers were synthetized at the “DNA Synthes” company (Moscow, Russia). To obtain DNA substrates for the primer extension reactions, the primers were 5′-labeled with γ-[^32^P]-ATP by T4 polynucleotide kinase (SibEnzyme, Novosibirsk, Russia) and annealed to the corresponding unlabeled template oligonucleotides at a molar ratio of 1:1.1, heated to 95 °C, and slowly cooled down to 20 °C. To purify the labeled locking DNA strand (used in the experiment with FEN1), unreacted γ-[^32^P]-ATP was removed on a MicroSpinTM G-25 column (Amersham Pharmacia Biotech, GE Healthcare, Chicago, IL, USA), and the labeled oligonucleotide was precipitated from the solution by 4% LiClO_4_ in acetone.

DNA substrates for strand displacement reactions were obtained by annealing 5′ [^32^P]-labeled primer, unlabeled template, and unlabeled downstream oligonucleotide at a molar ratio of 1:1:1. The sequences of the oligonucleotides used in this study are shown in Table 1.

### 4.3. Primer Extension Reactions

Primer extension reactions with PrimPol were carried out in 20 μL of reaction buffer containing 30 mM HEPES (pH 7.0), 8% glycerol, 0.1 mg/mL bovine serum albumin, 0.25–10 mM MgCl_2_ or MnCl_2_, 20 nM DNA substrate, 200 μM dNTPs, and 100 or 400 nM PrimPol. Some reactions were supplemented with 300 nM PolDIP2 or 20 nM RPA. Standard reactions with Pol β and Pol η were carried out in 20 μL of reaction buffer containing 30 mM HEPES (pH 7.4), 8% glycerol, 0.1 mg/mL bovine serum albumin, 10 mM MgCl_2_, 20 nM DNA substrate, 50 μM dNTPs, and 10 nM of polymerase. Reactions with FEN1 were carried out with 30 nM Pol β or 400 nM PrimPol and 400 nM FEN1. Locking oligonucleotides with 5′-phosphates was used in the experiments unless specified in figure legends.

Reactions were started by adding dNTPs and were incubated at 37 °C at different times as indicated in the figure legends. The reactions were terminated by the addition of 20 μL of loading buffer containing 95% formamide, 10 mM EDTA, and 0.1% bromophenol blue. DNA products were resolved on 21% or 30% denaturing PAGE and detected by phosphor imaging on Typhoon 9400 (GE Healthcare, Chicago, IL, USA). Experiments were repeated 3–4 times. To estimate the efficiency of strand displacement, the percentage of displaced DNA was calculated: The total intensity of bands of displaced DNA starting from +6 position (the band above the mark 23) was divided by the total intensity of all bands of displaced DNA and the +5 position band (marked as 23) corresponding to the pause site.

## Figures and Tables

**Table 1:** Oligonucleotides used in the study.

Primer	5′-CGGTATCCACCAGGTCTG-3′
34-mer DNA template	5′-GGCTTCATCGTTGTCXCAGACCTGGTGGATACCG-3′ X = G or 8-oxo-G
55-mer DNA template	5′-GACTACATTTCATCTGGCTTGGGCTTCATCGTTGTCGCAGACCTGGTGGATACCG-3′
Locking primer 1 (11-mer 5′-P-DNA)	5′-(P)ACGATGAAGCC-3′(P)—phosphate
Locking primer 2 (11-mer DNA)	5′-ACGATGAAGCC-3′
Locking primer 3 (32-mer 5′-P-DNA)	5′-(P)ACGATGAAGCCCAAGCCAGATGAAATGTAGTC-3′ (P)—phosphate
Locking primer 4 (32-mer DNA)	5′-ACGATGAAGCCCAAGCCAGATGAAATGTAGTC-3′
Locking primer 5 (32-mer 5′-P-RNA)	5′-(P)ACGAUGAAGCCCAAGCCAGAUGAAAUGUAGUC-3′ (P)—phosphate
Locking primer 6 (32-mer RNA)	5′- ACGAUGAAGCCCAAGCCAGAUGAAAUGUAGUC-3′

## Supplementary Materials

The following are available online at https://www.mdpi.com/1422-0067/21/23/9027/s1.

## Abbreviations

ssDNA	Single-stranded DNA
8-oxo-G	8-oxoguanine
