# Supplementary material for: Strand Displacement Activity of PrimPol
Source: Int J Mol Sci. 2020 Nov 27;21(23):9027. doi: 10.3390/ijms21239027 (PMC7729601; doi:10.3390/ijms21239027)
Supplement: Supplementary file 1 [file ijms-21-09027-s001.pdf]

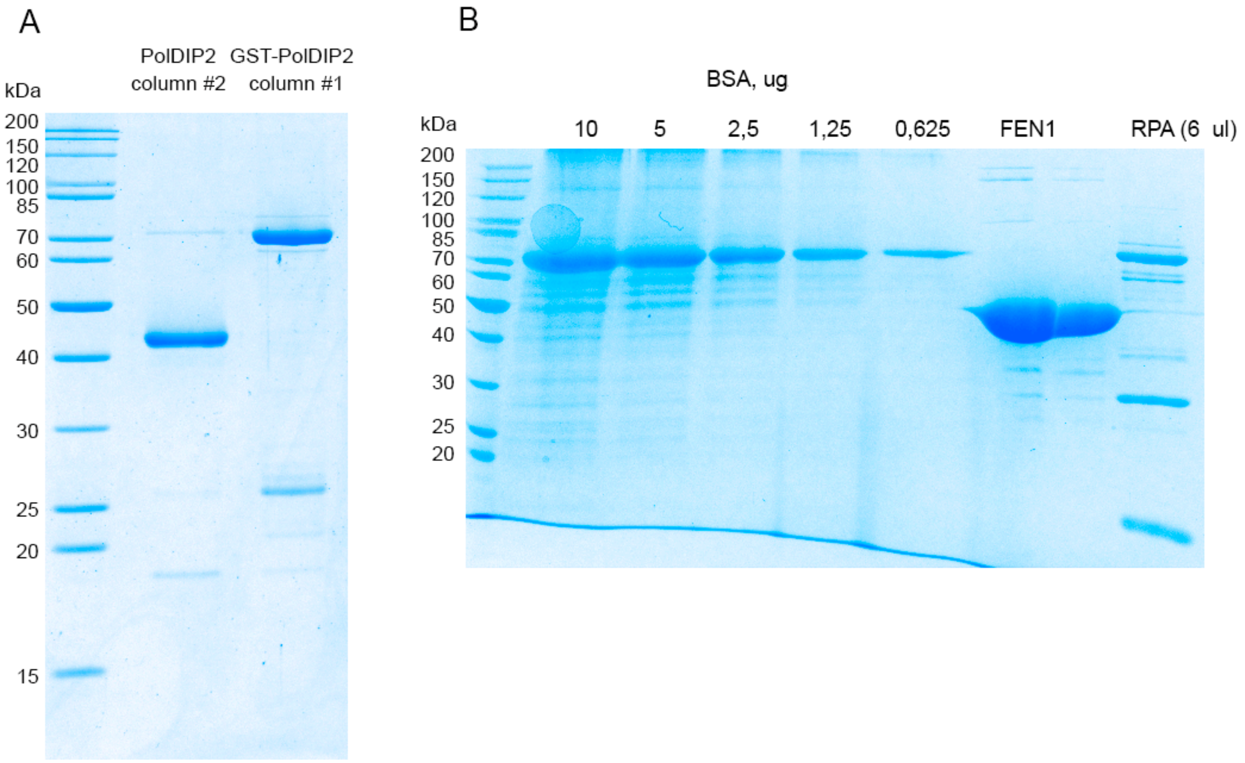

Supplementary figure 1. A. Preparation of PolDIP2 used in the study. B. Preparations of FEN1 and RPA used in the study.
